# Interactions and exchange of CO_2_ and H_2_O in coals: an investigation by low-field NMR relaxation

**DOI:** 10.1038/srep19919

**Published:** 2016-01-28

**Authors:** Xiaoxiao Sun, Yanbin Yao, Dameng Liu, Derek Elsworth, Zhejun Pan

**Affiliations:** 1 Coal Reservoir Laboratory of National Engineering Research Center of CBM Development & Utilization, China University of Geosciences, Beijing 100083, PR China; 2 Department of Energy and Mineral Engineering, Pennsylvania State University, University Park, PA 16802, USA; 3 CSIRO Energy Flagship, Private Bag 10, Clayton South, VIC 3169, Australia

## Abstract

The mechanisms by which CO_2_ and water interact in coal remain unclear and these are key questions for understanding ECBM processes and defining the long-term behaviour of injected CO_2_. In our experiments, we injected helium/CO_2_ to displace water in eight water-saturated samples. We used low-field NMR relaxation to investigate CO_2_ and water interactions in these coals across a variety of time-scales. The injection of helium did not change the *T*_2_ spectra of the coals. In contrast, the *T*_2_ spectra peaks of micro-capillary water gradually decreased and those of macro-capillary and bulk water increased with time after the injection of CO_2_. We assume that the CO_2_ diffuses through and/or dissolves into the capillary water to access the coal matrix interior, which promotes desorption of water molecules from the surfaces of coal micropores and mesopores. The replaced water mass is mainly related to the Langmuir adsorption volume of CO_2_ and increases as the CO_2_ adsorption capacity increases. Other factors, such as mineral composition, temperature and pressure, also influence the effective exchange between water and CO_2_. Finally, we built a quantified model to evaluate the efficiency of water replacement by CO_2_ injection with respect to temperature and pressure.

Carbon dioxide (CO_2_) is the predominant greenhouse gas in the atmosphere. As energy demand (a major cause of CO_2_ emissions) increases, annual CO_2_ emissions are expected to reach 20–35 Pg C y^−1^ by 2100 from the 1990 baseline emission rate of 5.5 Pg C y^−1^ 1. Geological sequestration of CO_2_ is considered to be a viable option to mitigate these effects; therefore, it is important to understand the long-term fate of CO_2_ in the subsurface in general, and in unmineable coals in particular^2^,^3^. Predictions concerning the efficiency of fluid replacement by CO_2_ injection in different types of coal require understanding of how the fluid is held in place and what factors might induce its release^4^. Reliable estimates of the interactions and exchange of CO_2_ and H_2_O in coals are needed for economic assessment of the viability of potential coal seams for enhanced coalbed methane recovery (ECBM).

CO_2_ in coalbeds is mainly in three states: as a free gas within pore spaces; dissolved in pore space liquids; or as a gas adsorbate bonded to the inner surfaces of coal micropores^5^. CO_2_ gas adsorbed into coal can displace some adsorbed methane and alter the adsorption capacity of the methane in coal^6^,^7^,^8^. As a consequence, the injection of CO_2_ in coalbeds can enhance the recovery of coalbed methane^9^,^10^. Most previous studies have focused on the CO_2_ gas sorption process^11^,^12^,^13^, and the results suggest that adsorption is the main trapping mechanism for CO_2_ storage in coal seams (accounting for about 95–98% of total storage). Silva *et al*. (2012) suggested a model for estimating the CO_2_ storage capacity in coal seams by using five parameters: volatile matter content, moisture, ash, pressure and temperature. However, the existence of water in coal reservoirs is not considered in this model. In general, previous studies have focused on understanding and quantifying the gas-coal interaction, but little attention has been paid to understanding the comprehensive influence of gas-coal-water interactions on CO_2_ storage in coal or ECBM.

In the context of reservoir engineering, coalbeds are naturally fractured and saturated with water. Because *in-situ* coal reservoirs commonly act as methane-coal-water systems, accurate predictions of CO_2_ sequestration capacity rely crucially on a comprehensive understanding of interactions among CH_4_, CO_2_ and water and of transport processes from fractures to pores across a variety of length- and time-scales^14^,^15^. With water molecules filling the voids, less space and surface area are available for gas flow and CO_2_ storage, respectively. Therefore, higher water content will markedly reduce the adsorption potential as water molecules take up the coal pore spaces that would otherwise have been available for CO_2_ adsorption^10^. Consequently, competitive adsorption exists between CO_2_ and water molecules in addition to that between methane and CO_2_. Some previous studies have investigated methane-coal-water interactions on the coal surface, such as contact angles and wetting behaviour in CO_2_–coal–H_2_O systems. Siemons *et al*. (2012)^16^ and Saghafi *et al*. (2014)^17^ measured the pressure dependence of the contact angle in a CO_2_–coal–H_2_O system and evaluated the role of wetting behaviour in CO_2_-ECBM production. However, surface interactions in a CO_2_–coal–H_2_O system have only a very limited influence on CO_2_ storage or ECBM. In contrast, interactions within pores in a CO_2_–coal–H_2_O system are extremely important for CO_2_ storage and ECBM. Interactions within pores are related not only to coal characteristics, such as composition, rank, porosity, permeability and physical adsorption capacity of coals^18^, but also to formation pressure and geothermal temperatures, which are extremely important. Day *et al*. (2011)^19^ observed the moisture loss during CH_4_/CO_2_ adsorption to moist coal in their work on swelling of moist coal. However, there have been no detailed discussions about inner interactions within CO_2_–coal–H_2_O systems. In this paper, we discuss the interactions and exchanges between CO_2_ and water within coal and propose a quantifying model for evaluating the processes of CO_2_ sequestration and CO_2_-ECBM.

Nuclear magnetic resonance (NMR) provides a fast, convenient and non-destructive method for detecting hydrogen-bearing fluids^20^. This technique has been used to determine various petrophysical characteristics such as porosity and permeability of the formation and viscosity and saturation of fluids in conventional reservoirs, and for well logging in petroleum exploration^21^,^22^,^23^. In coalbed methane exploration, NMR has also been used to characterise the porosity, pore geometry, pore connectivity and permeability of coal^20^. It has also been used to study methane adsorption and the migration of moisture in coals^24^,^25^. This paper is the first attempt to monitor interactions between CO_2_ and water in coals by using a series of low-field NMR measurements. In this study, gas and water exchange processes are followed as a function of time, temperature, pressure and coal properties using changes in the configurations of transverse relaxation time (*T*_2_) distributions of the water in the coal as a proxy. This process allows quantitative evaluation of the effects of coal properties, temperature and pressure on water-CO_2_ exchange, which is applicable for the successful operation and modelling of CO_2_ sequestration.

## Principle of NMR measurement

NMR theory has been discussed in detail in numerous articles (e.g., Howard *et al*.^26^, Kenyon *et al*.^27^ and Kleinberg *et al*.^28^). The underpinning principle of the method is that proton NMR transverse relaxation time (*T*_2_) is affected by bulk, diffuse and surface relaxation according to the basic characteristics of NMR measurements in rock, characterized by:


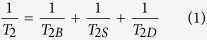


where the subscripts *B*, *S*, and *D* refer to bulk, surface, and diffuse relaxation, respectively. Diffusion relaxation is minimized in this study through the use of a homogeneous magnetic field and a Carr-Purcell-Meiboom-Gill pulse sequence (CPMG) with an echo spacing of less than 1 ms^29^. Bulk fluid relaxation is an intrinsic property of the fluid, which relaxes slowly, and signal peaks appear at longer relaxation times. Surface relaxation is rapid and is affected by the interaction of the fluid with the surface. Surface relaxivity and the ratio of the pore surface area to the pore volume are proportional to the surface relaxation and can be described by the following equation^30^:


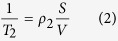


where 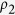 is the surface relaxivity representing the transverse relaxation strength and *S*/*V* is the surface-to-volume ratio relating to the size of the pore^31^.

Therefore, for a homogeneous internal field gradient, as used in this study, equation (2) becomes


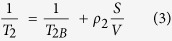


According to equation (3), protons in smaller pores and with high *S*/*V* values relax faster than those in larger pores. Therefore, the *T*_2_ of water hydrogen nuclei in coal is proportional to the pore radius. Consequently, the *T*_2_ distribution in coal samples reflects the water in different size pores, with the smallest pores having the shortest relaxation time and the largest pores having the longest relaxation time^25^. The total amplitude of the NMR signal is proportional to the fluid content of the rock as a result of NMR measuring only the amount of hydrogen in the rock^32^. In consequence, the total amplitude of the NMR signal serves as an indicator of the amount of water in the coal-hence its utility in this study. Neither CO_2_ nor helium contains mobile hydrogen protons and therefore neither will produce any signal in low-field NMR. Thus, the gas and water exchange behaviour is determined by measuring changes in the water signal in the coal samples.

### Sample Characterization

Eight block samples of coal were collected from underground mines of the Tarim, Ordos and Qinshui basins, China. All samples were carefully packed and then transported to the laboratory for experiments. The results of the vitrinite reflectance, maceral analyses and proximate analyses are listed in Table 1. The selected coals represent a broad range of coal ranks and lithotype compositions. These coals are bituminous to anthracite, with mean maximum vitrinite reflectance in oil (R_o_, %) ranging from 0.64% to 3.13%. Coal macerals are mainly characterized by intermediate to high vitrinite (62.5–95.4% volume) that corresponds to intermediate to low inertinite (0.2–32.4%), plus minor proportions of mineral matter (<13%) and trace amounts of liptinite (0–2.6%). The fixed carbon content of the coals ranges from 34.83% to 77.48%, the ash content of the coals ranges from 1.58% to 35.9% and the inherent moisture content ranges from 0.52% to 5.82%.

Each coal sample was crushed to form fragments with a diameter of ~1 cm and weight of ~1.5 g. All coal samples were placed in poly-teflon vials that could contain a maximum of ~22 g of coal. The nonmagnetic vials were placed in the sample cell. All the selected fragment samples were vacuum-dried in an oven at 80 °C for 6 h. After that, the 21 g coal samples were vacuumed for 48 h at room temperature and then saturated in distilled water for another 48 h. The coal samples were fully saturated with distilled water. The *T*_2_ spectrum of a 100% water-saturated coal sample is related entirely to the relaxation of water in the coal because the coal matrix is nonmagnetic.

## Methods

A series of four gas-water exchange experiments were completed. The experimental conditions of experimental suites A–D, including pressure, temperature, and injected gases, are given in Table 2. Coal segments with the same coal ID were taken from the same coal block. For series-A, three coals with different ranks (SJZ, DG and PL) were selected. After the pretreatment, water was removed from the coal surface and then the saturated-water coal segments were put into the sample cell. *T*_2_ measurements were then taken at different times after injecting helium gas into the sample cell, and the test pressure and temperature were kept constant for 48 hours. For series-B, eight coal samples were selected and CO_2_ was injected for 72 hours. For series-C and series-D, the SJZ and DS samples were used, and each sample was divided into five groups. Considering that high temperatures will result in the evaporation of water and affect the accuracy of the experimental data, the series-D experiments were completed at temperatures of 35 °C and 45 °C for only 48 hours.

## Results

### Liquid N_2_ adsorption analyses

In this study, the pore sizes measured in the coals were classified as either adsorption pores (<100 nm in diameter) or seepage pores (≥100 nm in diameter)^33^. The adsorption pores include micropores with diameters <10 nm and mesopores with diameters from 10 nm to 100 nm^34^. Liquid nitrogen adsorption experiments were mainly used for the analysis of adsorption pores.

The pore size distributions of the selected samples are given in Supplementary Fig. S1 and Supplementary Table 1. The total pore volumes of the selected samples varied from 1.012 to 22.376 × 10^−3^ mL/g, and the pore volumes of the adsorption pores varied from 0.771 to 20.635 × 10^−3^ mL/g. The adsorption pores were extremely well developed in sample DG, well developed in WTP, XG, DS and TCG, but poorly developed in PL, LY, and SJZ.

### Isothermal CO_2_ adsorption analyses

The CO_2_ adsorption isotherms of eight coals are shown in Supplementary Fig. S2, and the Langmuir volume and pressure are given by the as-received-base in Supplementary Table 2. The Langmuir volumes ranged from 27.19 to 52.61 m^3^/t and those of samples DG, DS, and WTP were distinctly higher than those of the other samples.

### Determination of the water amplitude index by NMR

The amplitude index (*AI*) was used to quantify the water content in coals using NMR. The *AI* is defined by the ratio of the water mass to the total *T*_2_ peak area. Bulk water with relaxation reagent masses ranging from 0.4326 to 2.5649 g was used to calculate water *AI* prior to the NMR experiments. As shown in Supplementary Fig. S3, the *T*_2_ spectra amplitude for bulk water increased with water mass. The *T*_2_ spectra were centred near 100 ms, which is less than the relaxation time (approximately 1000 ms) for bulk water. This is due to the influence of the relaxation reagent added to water to shorten the relaxation time of the bulk water. Note that the relaxation reagent has no influence on the total number of hydrogen atoms detected by low-field NMR.

The total *T*_2_ amplitude is plotted against the water mass in Supplementary Fig. S4. There is a linear relationship between total *T*_2_ amplitude and water mass. Therefore, we can calculate the water mass in the coals by the equation:


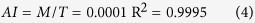


where *M* is the mass of water (g) and *T* represents the total amplitude of the measured *T*_2_ spectra.

## Discussion

### Relaxation characteristics of water in the coals

**Figure 1:**
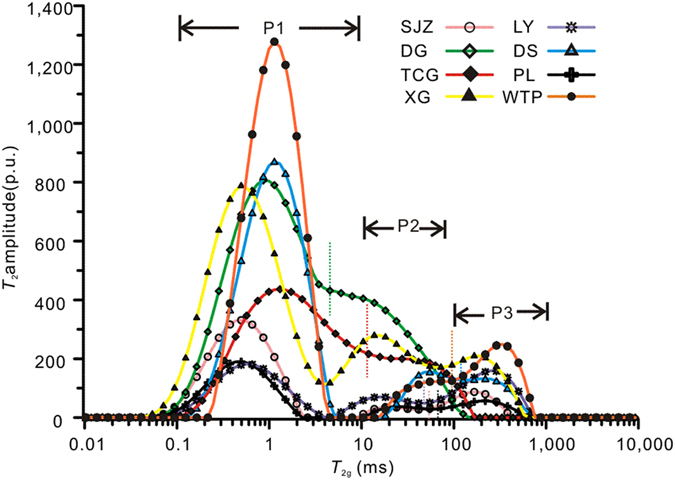
*T*_2_ spectra of water-saturated coal samples in series-B experiments (before injecting gas).

The *T*_2_ spectra of coal samples from series-B experiments are shown in Fig. 1. The *T*_2_ spectra distributions of samples of SJZ, PL, LY, DS, XG and WTP show three distinct peaks: the P1 peak centred at approximately 0.1–10 ms, the P2 peak at approximately 10–100 ms and the P3 peak at >100 ms (Fig. 1). Generally, the P3 peak is centred near the *T*_2_ range of 100–1000 ms, corresponding to the bulk water in the coal cleat and on the coal surface. The P2 peak results from the surface relaxation of water in the macropores (≥100 nm in diameter) of the coal as a result of a larger surface relaxation time. The P1 peak, which is attributed to surface relaxation at the pore walls, provides information about adsorbed water in pores with diameters <100 nm (i.e., the adsorption pores)^20^. Thus, the P1, P2, and P3 peaks represent “adsorbed water”, “macro-capillary water”, and “bulk water”, respectively. In contrast, for samples of DG and TCG, there are only “adsorbed water” and “macro-capillary water” peaks; the “bulk water” peak is negligible. The subbituminous coal TCG and high volatile bituminous coal DG contain no significant bulk water as a result of poor development of cleats to form P3 peaks compared to relatively high rank coals.

For DG and TCG samples, the well-connected bimodal distribution (P1 and P2 peaks) suggests that well-connected multi-scale pores exist, whereas a wide distribution represents multiple pore types in the coals. Liu *et al*.^35^ found that micropores in low-rank coals are open or semi-open in most cases, and these pores are connected by pore throats. In addition, the maceral composition is closely related to the pore distribution^36^. Duan *et al*.^37^ indicated that there is a complete and continuous pore system in inertinites, in which pores have a uniform shape and are open or semi-open in most cases. Therefore, samples DG and TCG, which have relatively low ranks and high inertinite content, show a multiple and well-connected pore type.

The *T*_2_ amplitude of adsorbed water (P1 peak) can be transformed into the adsorbed water mass using equation (4). The calculated adsorbed water mass is plotted against the mesopore and adsorption pore volume measured by N_2_ adsorption analysis (Supplementary Fig. S5). As shown in Supplementary Fig. S5 b, the adsorbed water mass is linearly correlated (R^2^ = 0.9336) with the mesopore volume. In contrast, the adsorbed water mass shows a logarithmic instead of linear relationship with increasing adsorption pore volume (Supplementary Fig. S5a). Thus, we assume that the adsorbed water mass measured by NMR is mainly contributed by water in the mesopores. Only some of the measured adsorbed water is contributed by the micropores because only limited micropores can be detected by low field NMR owing to the intrinsic precision limits of the NMR instrument used.

As shown in Fig. 1, the *T*_2_ distributions of adsorbed water are different for different coals. For simplicity, in this study, the terms “lower-rank”, “medium-rank” and “higher-rank” coals refer to samples DG, XG and TCG (with Ro of 0.64%, 1.12% and 0.94%), PL, LY and SJZ (with Ro of 1.67–2.57%), and DS and WTP (with Ro of 3.0% and 3.13%), respectively. The lower-rank and higher-rank coals have high adsorbed water contents, whereas the medium-rank coals have low adsorbed water content. During coalification, the number of adsorption pores initially decreases and then increases as coal rank increases. For the lower-rank coals, mesopores and micropores are abundant. As a consequence of polycondensation of coal molecules, the number and diameters of mesopores decrease with coalification, and the micropore structure varies only slightly^38^. For the higher-rank coals, the number of mesopores increases, probably owing to the increase in gas generation from the coal. The original micropores are enlarged into mesopores by gas generation, which is beneficial for gas diffusion and transport^39^.

### Water-helium gas interactions in the coals

**Figure 2:**
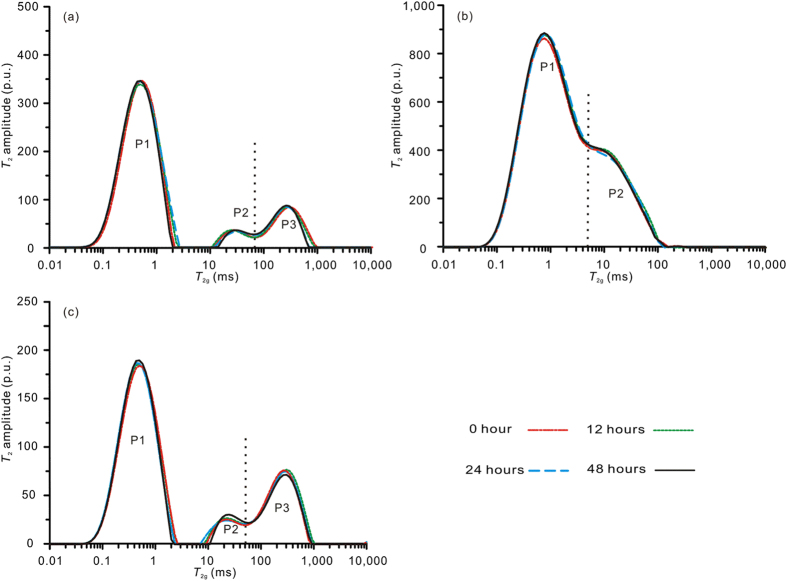
*T*_2_ spectra of coals in series-A experiments at different times after helium gas injection compared with *T*_2_ spectra before gas injection (a-SJZ; b-DG; c-PL).

Three water-saturated coals were injected with helium gas in the series-A experiments. The *T*_2_ spectra of coals measured at different times after gas injection are shown in Fig. 2 for comparison with the *T*_2_ spectra of coals measured prior to injection (0 hours after gas injection). The three samples exhibit little change after the injection of helium gas. Thus, pressurized non-adsorptive helium exerts little influence on the water distribution in the coals. Consequently, there is no interaction between water and helium gas in the coals.

### Water-CO_2_ gas interactions in the coals

**Figure 3:**
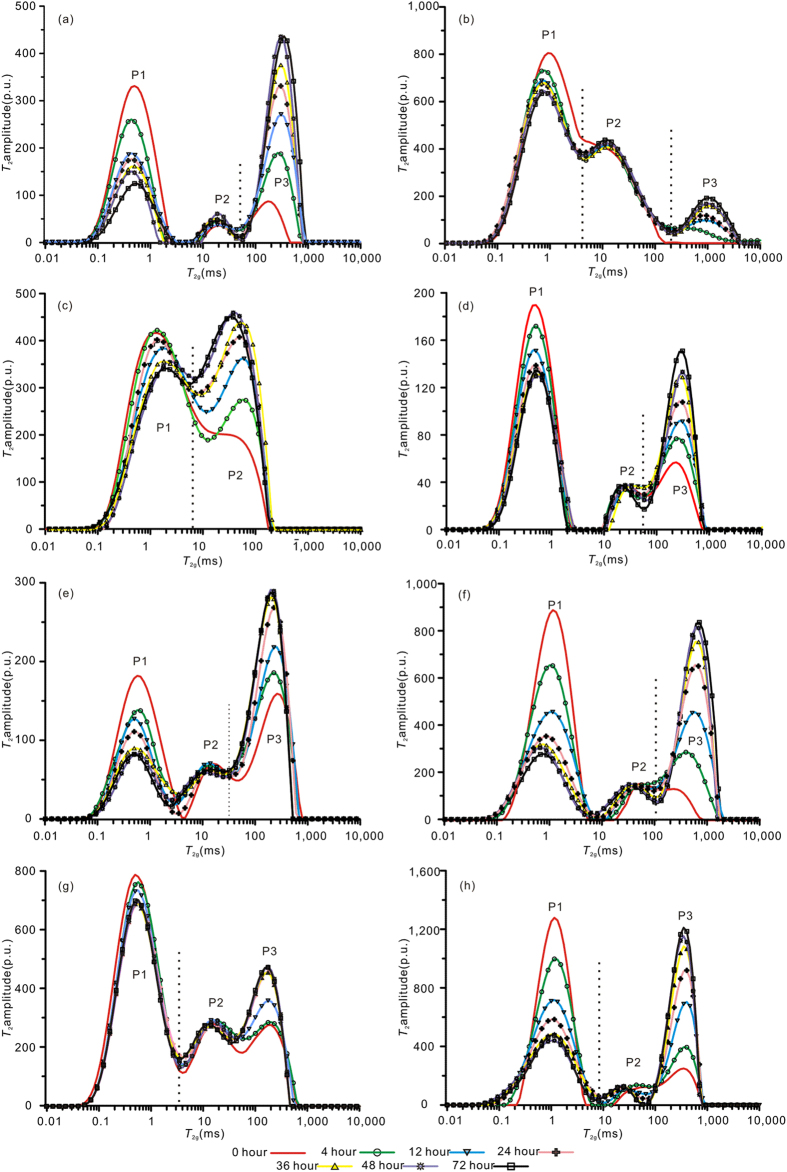
*T*_2_ spectra of samples in series-B experiments at different times after CO_2_ gas injection compared with *T*_2_ spectra before gas injection (a-SJZ; b-DG; c-TCG; d-PL; e-LY; f-DS; g-XG; h-WTP).

The *T*_2_ spectra of eight water-saturated coal samples were measured at different times after CO_2_ gas injection, as shown in Fig. 3. Unlike the *T*_2_ spectra of coal injected with pressurized helium, the *T*_2_ spectra of all samples change with CO_2_ residence time. For all coals, the adsorbed water peaks decrease as the bulk water peaks or macro-capillary water peaks increase (sample TCG); these changes are rapid at 0–36 hours and then slow at 36–72 hours. To explain this, we assume that the gas molecules diffuse through and/or dissolve into the capillary water to access the coal matrix interior after CO_2_ injection. In addition, CO_2_ gas molecules that are adsorbed to the surface of the coal micropores and mesopores will promote desorption of some water molecules from the coal. As a result, the replaced water molecules will migrate from the coal pores and coalesce into the bulk water. Thus, the interaction between adsorbed water and CO_2_ was confirmed by the series-B experiments.

### Comparison of the water-CO_2_ exchange effect in different coals

**Figure 4:**
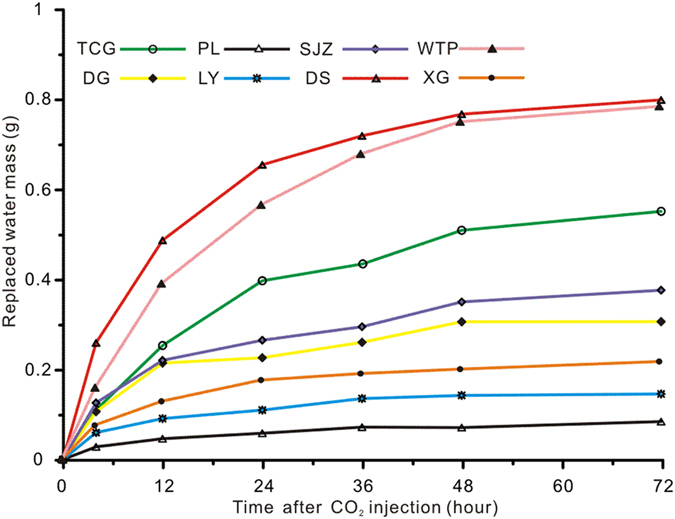
Increased mass of replaced water after CO_2_ injection (series-B experiment).

Different samples show different patterns of water replacement (Fig. 3). In this study, we assume that the exchange process reaches equilibrium after 48-hours of replacement – this is inferred from the small change in the *T*_2_ spectra of samples between 48 to 72 hours. We quantified the exchange and migration of water in the coals when the interaction process equilibrated. There are two methods for determining the quantity of water replaced by CO_2_ injection: measurement of the mass loss of adsorbed water or measurement of the complementary mass increase of macro-capillary water and bulk water. We used the latter method because the change in adsorbed water content in the micropores cannot be detected owing to limitations of the NMR instrument. Figure 4 shows the mass increase of replaced water after CO_2_ injection. For all samples, the mass of replaced water changes rapidly until ~24 h, and then the rate slows to zero thereafter. The exchange is complete after ~48 h of injection, when the exchange between gas and water reaches equilibrium.

**Figure 5:**
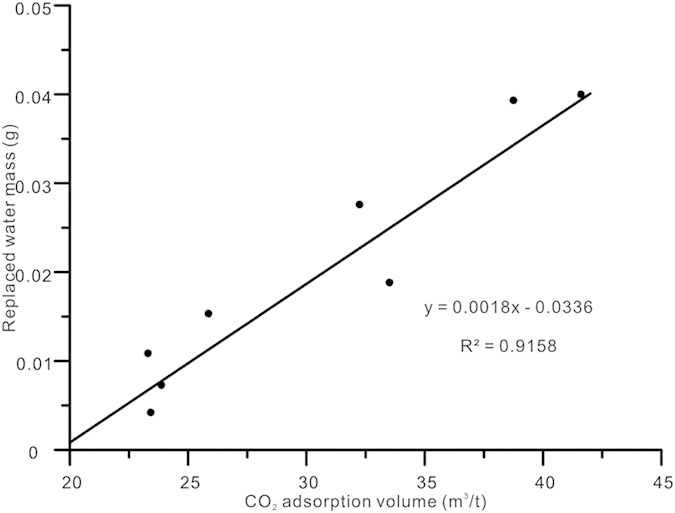
Relationship between CO_2_ adsorption volume at 25 °C and 4.5 MPa and the final replaced water mass per gram of coal after CO_2_ injection.

The quantity of water replaced by CO_2_ is related to the CO_2_ adsorption capacity of coals. In Fig. 5, the replaced water mass after 72 hours of injection is taken as the final replaced water mass. The Langmuir adsorption volume of CO_2_ at 25 °C and 4.5 MPa is used to determine the CO_2_ adsorption capacity of the coal samples. The replaced water mass shows a positive linear correlation with the Langmuir adsorption volume of CO_2_ – higher CO_2_ adsorption capacity yields greater replaced water mass. This suggests that the exchange between water and CO_2_ is mainly affected by CO_2_ adsorption capacity.

Interestingly, the CO_2_ adsorption isotherms of sample LY and sample PL are almost identical (Supplementary Fig. S2). However, the exchange capacity of sample LY is larger than that of sample PL. The reason for this may be the significant difference in composition and mineral content between the two samples. According to the results of coal proximate and maceral composition analyses, the ash yield of sample PL is as high as 32.15%, and clay acts as the main mineral component in the sample. In contrast, LY has low ash yields of 17.66%, and the main mineral component of the coal is pyrite. Compared with other minerals, clay may provide additional gas sorption capacity owing to its high internal surface area^40^. Clay minerals are hydrophilic and water can be easily adsorbed onto clay mineral surfaces, thereby reducing the gas sorption capacity of coal. Additionally, water molecules have stronger affinity than CO_2_ for the clay surface^40^,^41^. Thus, it is difficult for CO_2_ molecules to replace water molecules in clay layers owing to the strong adsorption of water onto the clay surfaces. Sample PL has higher clay mineral content than LY, which is the main reason for the lower water replacement in PL than in LY. Similarly, samples SJZ and TCG have similar adsorption capacities, but large differences in terms of replaced water mass. This may also be due to the different mineral compositions of the samples. Thus, the water-CO_2_ exchange process is also influenced by clay matter content in the coal. Furthermore, permeability, porosity, wettability, and other coal properties can influence the CO_2_–water exchange process^10^,^42^, and these influences will be investigated in future work.

### Effect of temperature and pressure on water-CO_2_ exchange

Experimental series C and D were carried out to investigate the effects of pressure and temperature on the exchange between adsorbed water and CO_2_. Supplementary Fig. S6 and Supplementary Fig. S7 show changes in the distribution of water in samples SJZ and DS with increased CO_2_ injection pressure, and Supplementary Fig. S8 shows the change in the replaced water mass after CO_2_ injection at 2.5, 3.5, 4.5, and 5.5 MPa. The adsorbed water peak decreases and bulk water peak increases as a result of CO_2_ injection, which is right for experiments at all gas pressures. Moreover, the amplitude of the decrease/increase varies with pressure: higher pressures yield greater replaced water mass (Supplementary Fig. S8). According to gas adsorption theory, gas adsorption is directly proportional to gas pressure at lower pressure. Coal adsorption capacity increases with pressure, and thus CO_2_ molecules can displace many more adsorbed water molecules as gas pressure increases.

The effect of temperature on the exchange process is shown in Supplementary Figs. S9, S10 and S11. Supplementary Figs. S9 and S10 show the distribution of water in the coals after CO_2_ injection at different experimental temperatures. The adsorbed water decreases with an increase in the volume of bulk water after CO_2_ injection. Supplementary Fig. S11 shows the change in the mass of replaced water after CO_2_ injection at different temperatures in samples SJZ and DS. The figure indicates a negative correlation between the replaced water mass and temperature: the final replaced water mass decreases as temperature increases. Adsorption is an exothermic process; therefore, increased temperature exerts a negative effect on CO_2_ adsorption on coal, and thus reduces the effective exchange between water and CO_2_. Hence, higher temperatures yield stronger negative effects.

Coal adsorption capacity is a function of the physical properties of temperature, pressure and adsorption medium^43^:


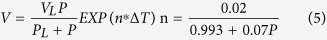


where *V*_*L*_ is the Langmuir volume, m^3^/t; *P*_*L*_ is the Langmuir pressure, MPa; *V* is the adsorption volume, m^3^/t; *P* is the gas pressure, MPa; and *ΔT* is the difference between the experiment temperature and coalbed temperature, °C.

According to equation (5), we can calculate the empirical adsorption volume under different experimental conditions (pressure and temperature). The experimental conditions of the series of experiments B, C and D for samples SJZ and DS, including temperatures and pressure (25 °C, 4.5 MPa; 35 °C, 4.5 MPa; 45 °C, 4.5 MPa; 25 °C, 2.5 MPa; 25 °C, 3.5 MPa; 25 °C, 5.5 MPa), were used to obtain the six adsorption volumes.

**Figure 6:**
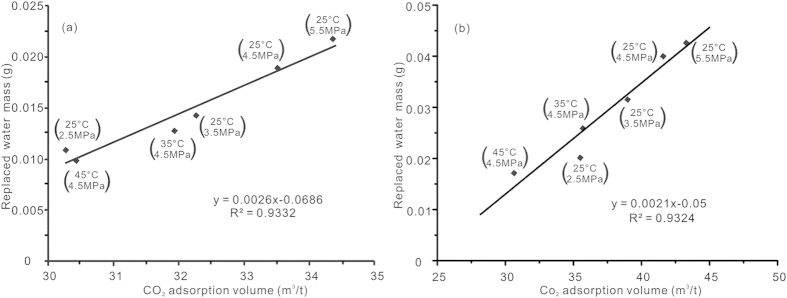
Relationship between CO_2_ adsorption volume and replaced water mass per gram of coal under different experiment temperatures and pressures (a-SJZ; b-DS).

The calculated adsorption volumes were plotted against the final replaced water mass, as shown in Fig. 6. A linear correlation was found between the adsorption volume and the replaced water under different experimental conditions for samples DS and SJZ. Thus, we can obtain relations fitting the replaced water mass and adsorption volume of samples SJZ and DS, respectively:


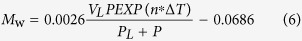



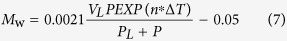


where *M*_w_ is the maximal replaced water mass per gram of coal, g; *V*_*L*_ is the Langmuir volume, m^3^/t; *P*_*L*_ is the Langmuir pressure, MPa; *P* is the gas pressure, MPa; Δ*T* is the difference between the experiment temperature and coalbed temperature, °C. The goodness-of-fits are 0.93 for both equations (6) and (7), indicating that the fits are excellent for the two samples (DS and SJZ).

Equations (6) and (7) can be combined into a universal equation:


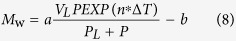


where *a* and *b* are defined as the replacement efficiency parameters that can be used to characterize the efficiency of water replacement by CO_2_ injection.

If we assume that samples SJZ and DS represent two different coal reservoirs for CO_2_ injection, then equations (6) and (7) can be used to quantify the replaced water mass of the coal reservoirs of SJZ and DS. Similarly, equation (8) provides a universal model to evaluate the efficiency of water replacement by CO_2_ injection with respect to different reservoirs. For application purposes, the input of the model includes the CO_2_ adsorption parameters of the coal (the *V*_L_ and *P*_L_), and the replacement efficiency parameters. The former are derived from an isothermal CO_2_ adsorption measurement, whereas the latter can be obtained from an NMR water-replacement experiment.

*a* and *b* are two variables in equation (8). Variable *a* is located in the molecules of the equation, and it can be merged with V_L_ in equation (8). Thus, the physical meaning of *a* is similar to V_L_. We assume that *a* is a parameter representing the effect of adsorption capacity on the exchange process: larger values of *a* yield greater replacement effects. Variable *b* may reflect the effect of other coal properties on the exchange process. These coal properties include organic and inorganic composition, porosity, permeability and gas/water wettability of coal. Note that the discussion about the physical meaning of parameters *a* and *b* is based only on our limited experimental results on samples SJZ and DS, and further related research is still needed to confirm these speculations.

### Reproducibility and uncertainties of the experiments

To evaluate the reproducibility or uncertainty of the experiments, we chose samples SJZ and DS to repeat series-B experiments at a pressure of 4.5 MPa and temperature of 25 °C, respectively. Two groups of reproducibility experiments were conducted for each selected coal sample (Supplementary Fig. S12). The results of samples DS and SJZ were compared with those of experiment-B to estimate the uncertainties of the experimental data, as shown in Supplementary Fig. S13.

Supplementary Tables 3 and 4 show the difference between the results of the reproducibility experiments and series-B experiments at different times after CO_2_ injection. The absolute deviation between the two sets of data is less than 0.05 g for sample SJZ, and less than 0.08 g for sample DS at different times after CO_2_ injection. Except for the first experimental point (four hours after CO_2_ injection), which has a large relative deviation mainly owing to unstable condition as the gas injection, the average relative deviation is <8.2% for sample SJZ and <4.2% for sample DS. Thus, we can reasonably assume that the NMR experiments on the interactions of water and CO_2_ in coals are repeatable for all other samples.

It should be noted that some uncertainties exist in our experiments. The first uncertainty is related to the sample size. Because effective diffusivities increase with decreasing particle size^44^, any difference in sample size may change the gas diffusion rate and the interaction rate between CO_2_ and water molecules at the beginning of the experiments. The diffusion rate affected by particle size leads to a greater difference in replaced water between different experiments at the beginning of the exchange process. However, when the process reaches equilibrium, the sample size does not affect the coal adsorption capacity^45^,^46^, and the effect on water-CO_2_ interaction will be greatly reduced. Secondly, coals have high heterogeneity in organic or inorganic compositions^47^; thus the adsorption capacity of coals is different for coals even with the same coal rank but different coal composition. Therefore, coal heterogeneity can also create additional uncertainty in the experiment results. Finally, the limitations of the NMR apparatus can also reduce the precision of the experimental results.

## Conclusions

In our experiments, we injected helium or CO_2_ to displace water in eight water-saturated samples of bituminous coal and anthracite. The injection of helium did not change the *T*_2_ spectra of the coals. In contrast, the *T*_2_ spectra peak of micro-capillary water gradually decreased and those of the macro-capillary and bulk water increased with time after the injection of CO_2_. We assume that the CO_2_ molecules diffuse through and/or dissolve into the capillary water to access the coal matrix interior, which promotes desorption of some water molecules from the surface of the coal micropores and mesopores. Thus, the adsorbed water in coals can be replaced by CO_2_, but not by the helium molecules.

The replaced water mass exhibits a positive linear correlation with the Langmuir adsorption volume of CO_2_ — higher CO_2_ adsorption capacity yields greater replaced water mass. Apart from the CO_2_ adsorption capacity, decreasing temperature and increasing pressure can enhance the effective exchange between water and CO_2_. Coal with relatively low clay matter content is favourable for the exchange between water and CO_2_.

Finally, we have built a quantitative model to calculate the water mass replaced by injection of CO_2_ at different pressures and temperatures. Using the model, the effects of coal properties, temperature and pressure on water and CO_2_ exchange can be evaluated and applied in the targeting of coal seams for CO_2_ sequestration.

## Additional Information

**How to cite this article**: Sun, X. *et al.* Interactions and exchange of CO_2_ and H_2_O in coals: an investigation by low-field NMR relaxation. *Sci. Rep.* **6**, 19919; doi: 10.1038/srep19919 (2016).

## Supplementary Material

Supplementary Information

Supplementary Dataset 1

Supplementary Dataset 2

Supplementary Dataset 3

Supplementary Dataset 4

Supplementary Dataset 5

Supplementary Dataset 6

Supplementary Dataset 7

Supplementary Dataset 8

Supplementary Dataset 9

Supplementary Dataset 10

Supplementary Dataset 11

## Figures and Tables

**Table 1:** Coal rank, maceral composition and proximate analysis of the selected coal samples.

Sample ID.	Coal basin	Coal mine	Coal seam	*R*_O_^a^ (%)	Maceral and mineral (vol. %)					Proximate analysis (wt.%, dry)		
					*V*^b^	*I*^b^	*L*^b^	MM^b^		*M*_ad_^c^	*A*_ad_^c^	*F*_cad_^c^
					(%)	(%)	(%)	P^b^	C^b^	(%)	(%)	(%)
								(%)				
TCG	Tarim	Tiechanggou	1#	0.64	63.2	31.4	2.6	0.1	2.7	2.72	3.32	34.83
DG	Tarim	Donggou	1#	0.94	67.9	27	2	0	3.1	5.82	1.58	48.83
XG	Tarim	Xigou	2#	1.12	62.5	32.4	0.4	0.2	4.7	4.85	3.38	77.48
PL	Ordos	Panlong	3#	1.67	87.8	5.6	0	0.4	6.2	0.52	32.15	53.77
LY	Ordos	Long yuan	11#	2.1	85.4	1.6	0	7.2	5.8	0.56	17.66	42.46
SJZ	Qinshui	Shenjia zhuang	3#	2.57	88.9	0.9	0	0	10.2	1.27	9.17	56.51
DS	Qinshui	Duanshi	3#	3.0	95.4	0.2	0	0.1	4.3	2.14	21.25	62.94
WTP	Qinshui	Wangtaipu	15#	3.13	89.7	6.4	0	0.5	3.4	1.83	35.9	58.89

^a^Mean maximum vitrinite reflectance in oil.

^b^*V*, *I*, and *L* represent the volume percentages of vitrinite, inertinite and liptinite in coal maceral composition, respectively. MM is the volume percentage of minerals on the dry base, P represents the volume percentages of pyrite and C represents clay and other minerals.

^c^*M*_ad_, *A*_ad_ and *F*_cad_ represent air-dry-based moisture content, ash yield and fixed carbon content, respectively.

**Table 2:** Experimental conditions for the four series of gas and water exchange experiments.

Experimental Series	Sample	Temperature (°C)	Pressure (MPa)	Gas injection
A	SJZ, DG, PL	25	4.5	Helium
B	SJZ, DG, PL, TCG, LY, DS, XG, WTP	25	4.5	CO_2_
C	SJZ, DS(three subsamples)	25	2.5, 3.5, 5.5	CO_2_
D	SJZ, DS (two subsamples)	35, 45	4.5	CO_2_
