# Supplementary material for: Interactions and exchange of CO2 and H2O in coals: an investigation by low-field NMR relaxation
Source: Sci Rep. 2016 Jan 28;6:19919. doi: 10.1038/srep19919 (PMC4730140; doi:10.1038/srep19919)
Supplement: Supplementary Information [file srep19919-s1.pdf]

Interactions and exchange of CO_2 and H_2O in coals: an investigation by low-field NMR relaxation

Xiaoxiao Sun, Yanbin Yao*, Dameng Liu, Derek Elsworth, Zhejun Pan

Supplementary Figures

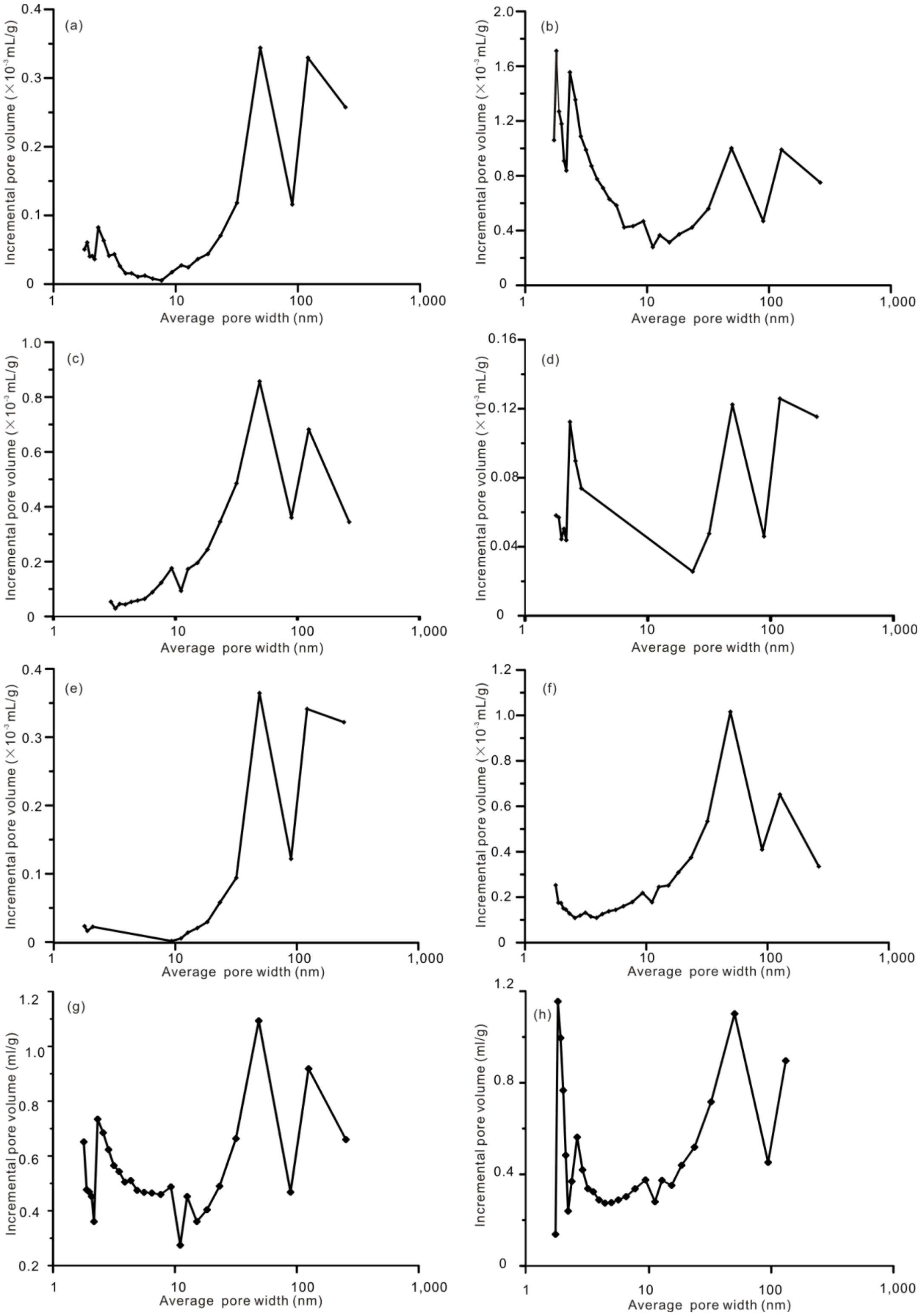

Supplementary Fig. S1. Adsorption pore distribution recovered from N_2 gas isotherm adsorption (a-SJZ; b-DG; c-TCG; d-PL; e-LY; f-DS; g-XG; h-WTP)

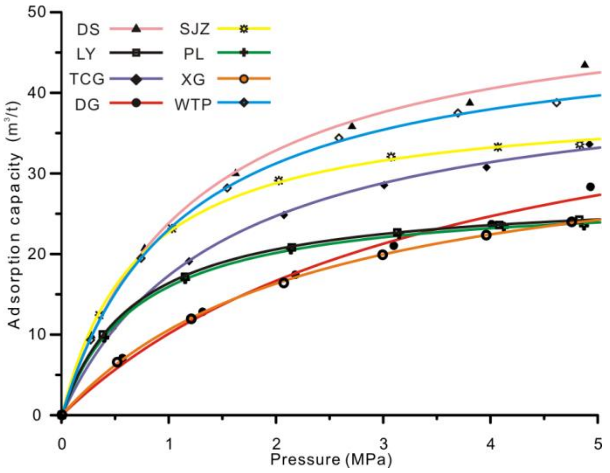

Supplementary Fig. S2. CO_2 isothermal adsorption of different rank coal samples for experiments at 25 ℃ in the pressure range 0- 4.9 MPa

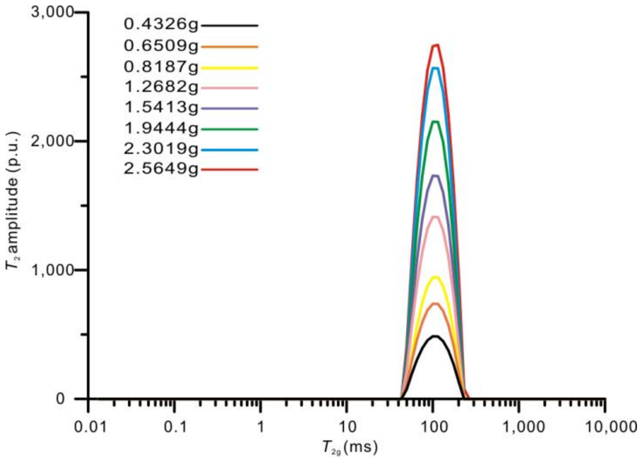

Supplementary Fig. S3. T_2 spectra of bulk water with masses ranging from 0.4326 to 2.5649g

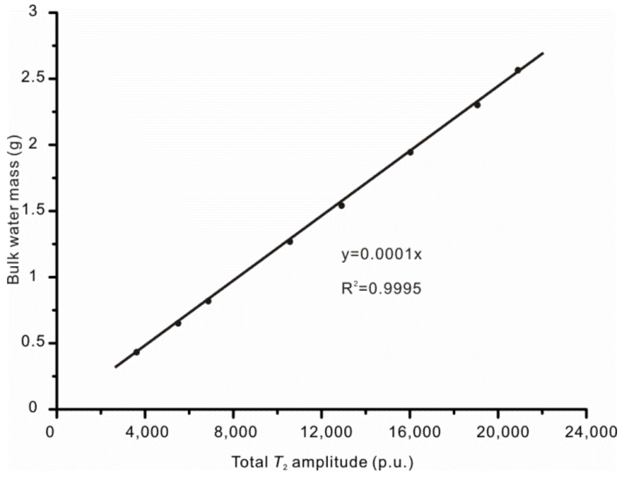

Supplementary Fig. S4. Mass of water versus total amplitude of water

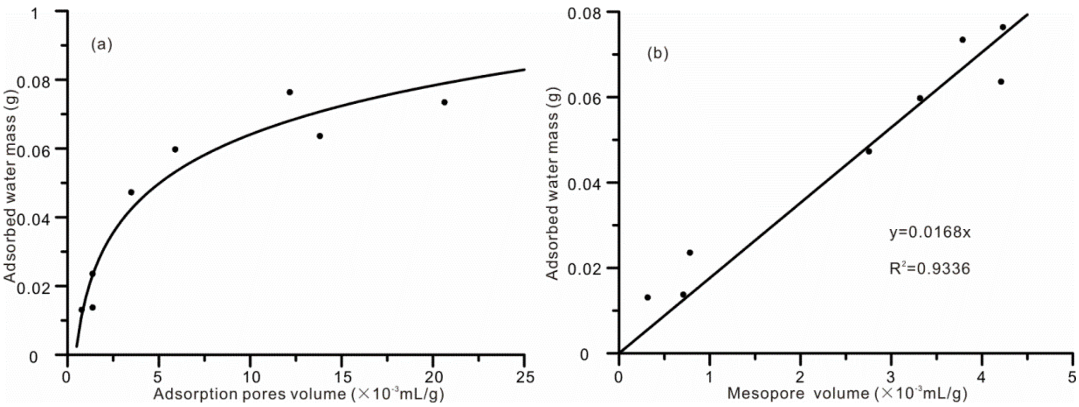

Supplementary Fig. S5. Relationship between adsorbed water mass per gram coal measured by NMR and (a) adsorption pore volume, (b) mesopore volume measured by N_2 gas isotherm adsorption

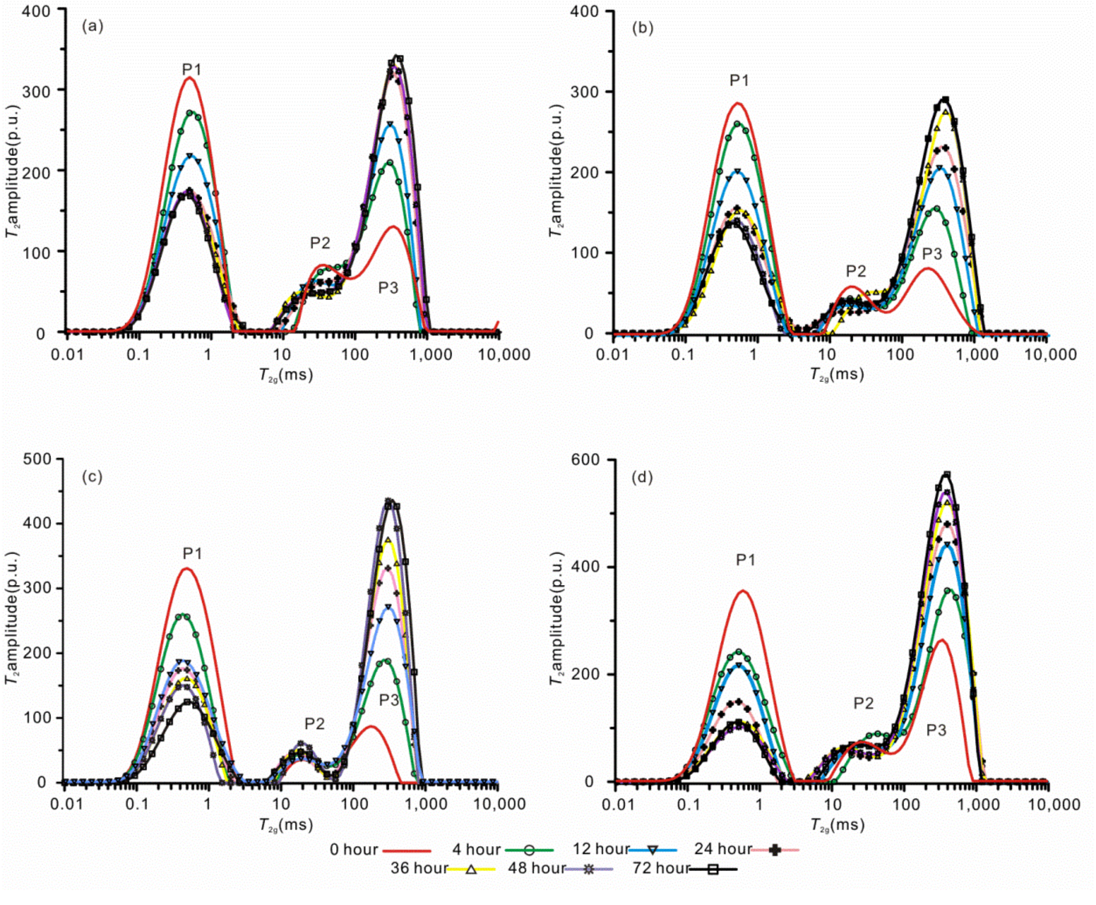

Supplementary Fig. S6. The change of T_2 spectra of coal sample SJZ after CO_2 injection, at different CO_2 injection pressures (a-2.5 MPa; b-3.5 MPa; c-4.5 MPa; d-5.5 MPa, where c is recovered from experiment B)

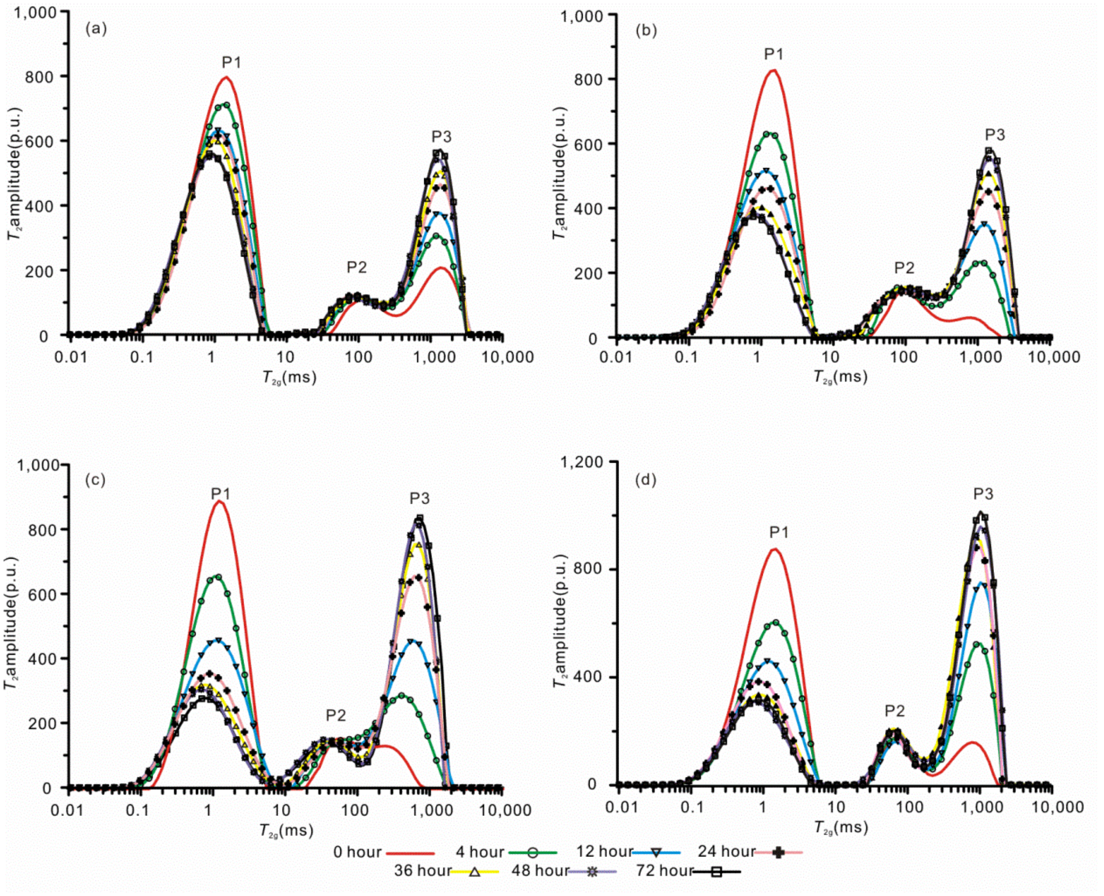

Supplementary Fig. S7. The change of T_2 spectra of coal sample DS after CO_2 injection, at different CO_2 injection pressures (a-2.5 MPa; b-3.5 MPa; c-4.5 MPa; d-5.5 MPa, where c is recovered from experiment B)

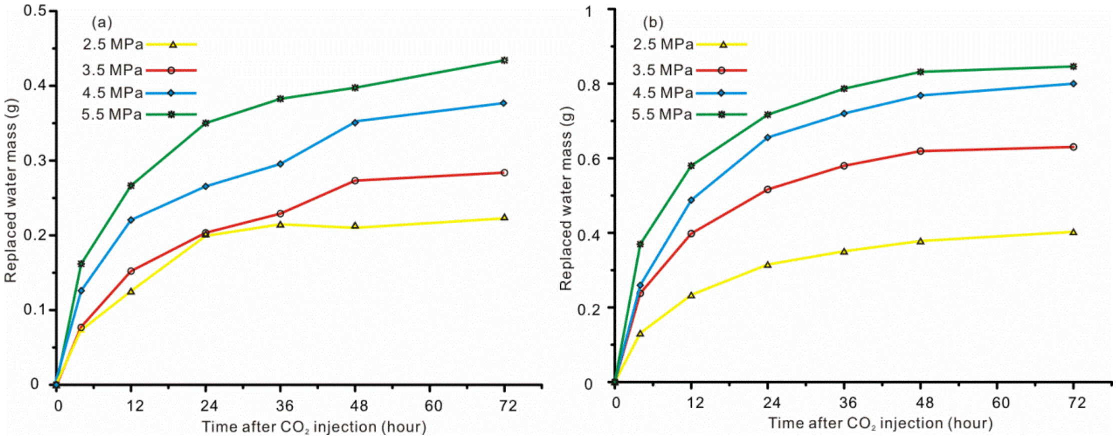

Supplementary Fig. S8. Changes of replaced water mass at different CO_2 pressures, (a) sample SJZ and (b) sample DS

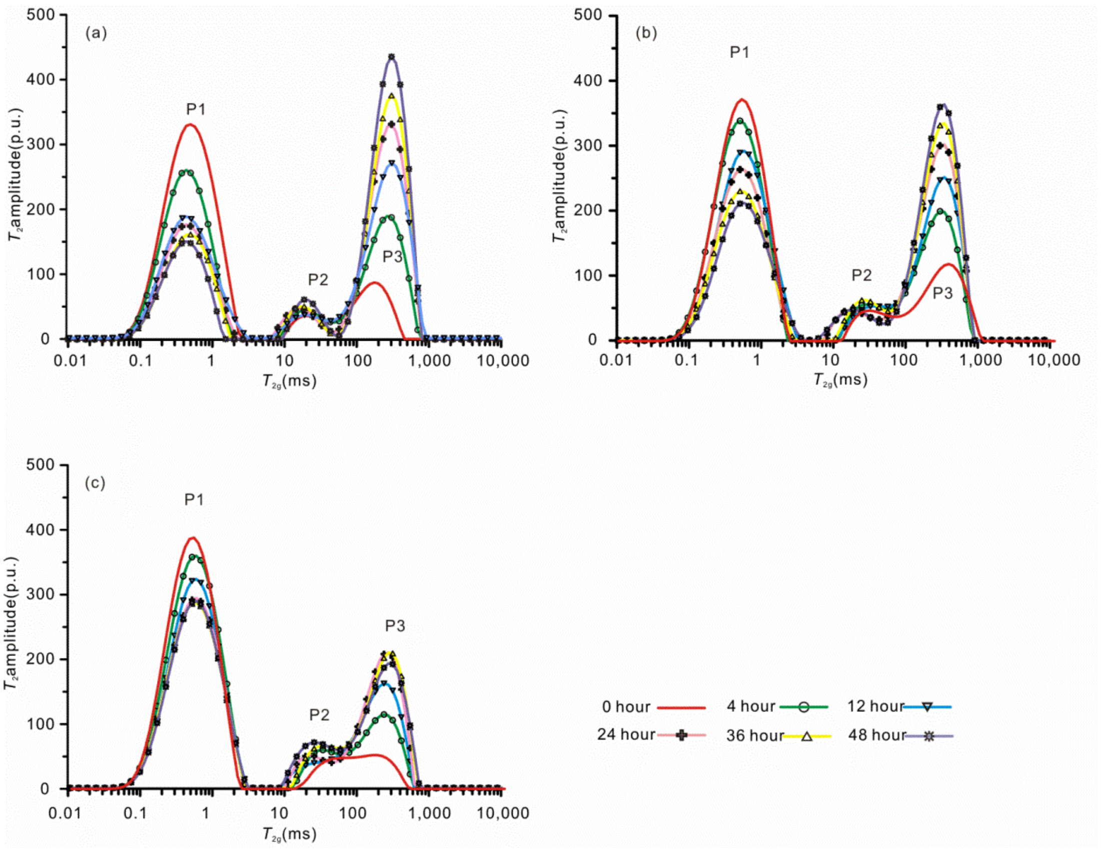

Supplementary Fig. S9. The change in T_2 spectra after CO_2 injection, at different temperatures (a-25 ℃; b-35 ℃; c-45 ℃, where a is recovered from experiment B), sample SJZ

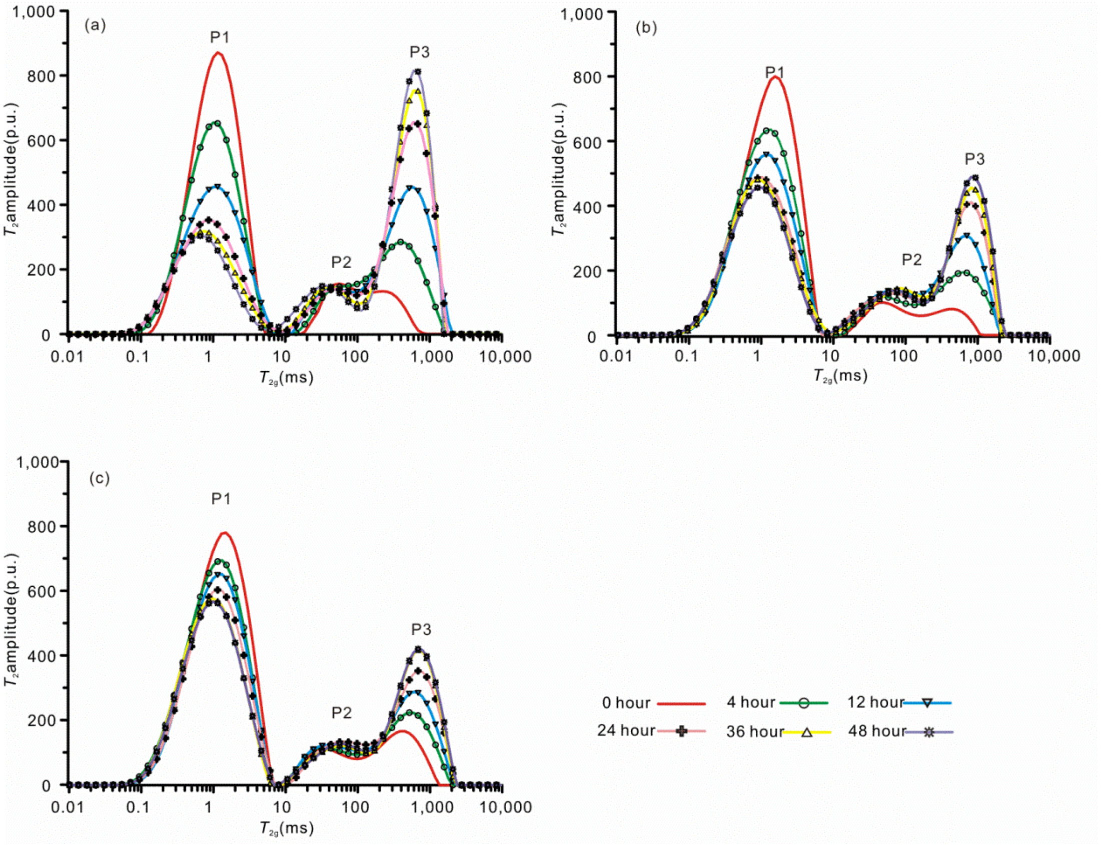

Supplementary Fig. S10. The change in T_2 spectra after CO_2 injection, at different temperatures (a-25 ℃; b-35 ℃; c-45 ℃, where a is recovered from experiment B), sample DS

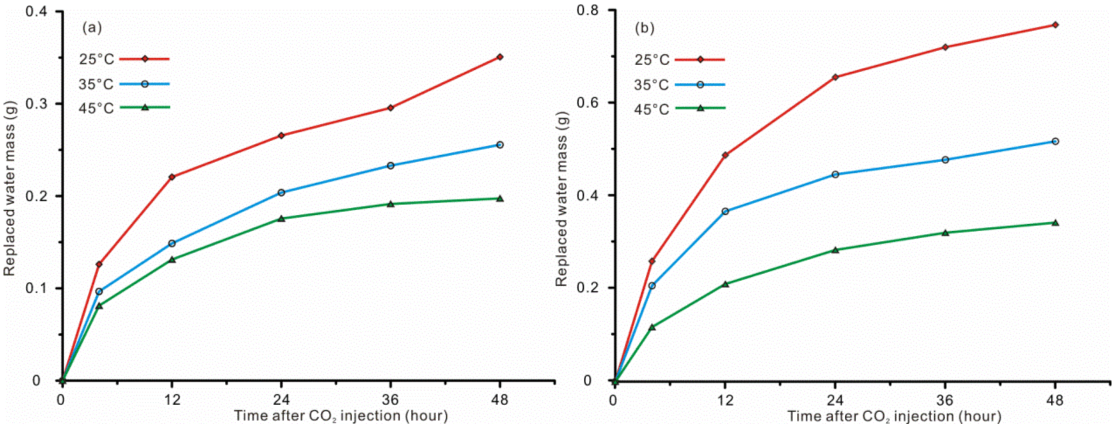

Supplementary Fig. S11. The change of replaced water mass after CO_2 injection, at different experimental temperatures for sample (a) SJZ and (b) DS

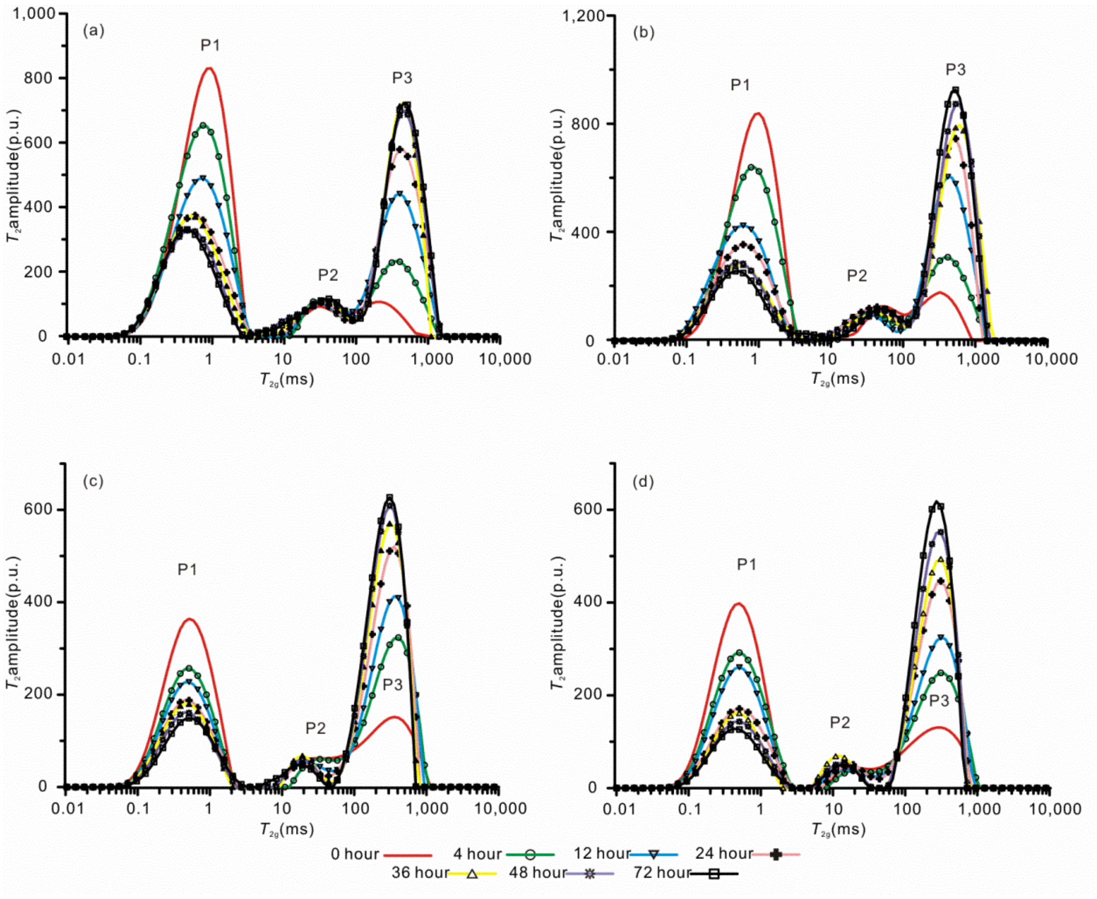

Supplementary Fig. S12. Reproducibility experiments for evaluating replaced water mass by CO_2-injection (a, b -DS and c, d- SJZ)

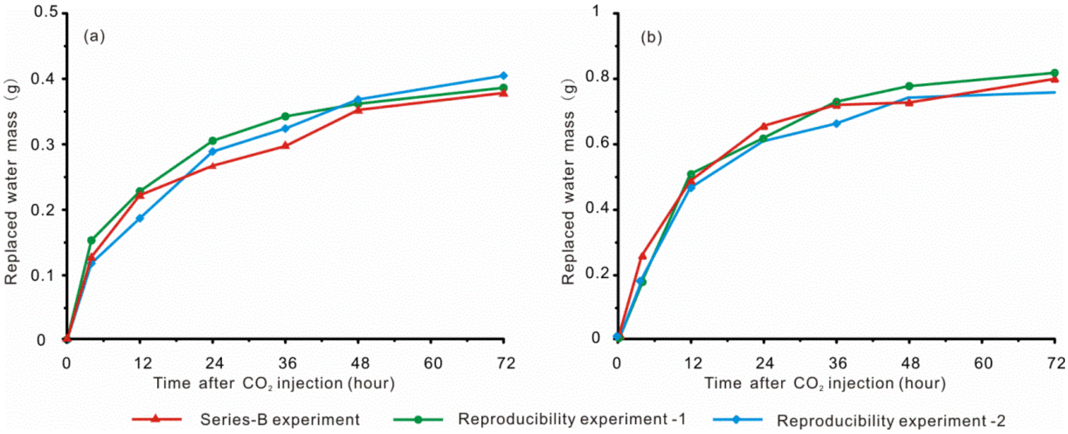

Supplementary Fig. S13. Increase mass of the replaced water after CO_2 injection, results of reproducibility experiments and series-B experiment (a-SJZ, b-DS)

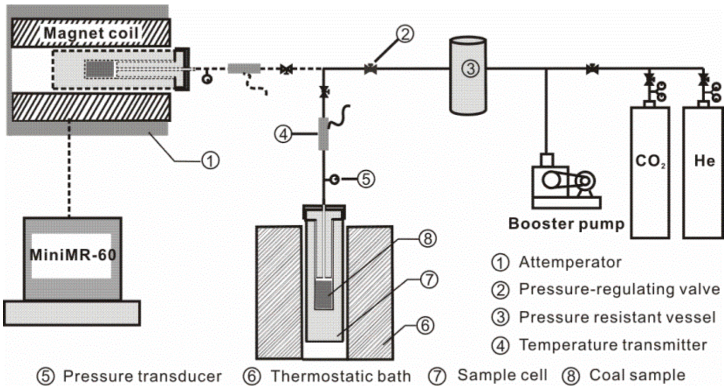

Supplementary Fig. S14. Experimental apparatus for gas and water exchange in coals at different temperature and pressure. The thermostatic bath apparatus is designed to maintain a constant temperature and the attemperator on the surface of the magnet coil maintains temperature when T_2 spectra are measured.

Supplementary Tables

Sample No		TCG	DG	PL	LY	SJZ	DS	XG	WTP
Total pore surface area (m^2/g)		0.970	36.618	0.539	0.394	1.596	9.485	15.362	53.097
Average pore diameter (nm)		18.628	2.843	18.522	14.928	7.545	6.943	4.314	4.007
Pore volumes($\times 10^{-3}$mL/g)									
	Micropores (<10nm)	0.735	16.849	0.456	0.663	0.587	2.576	9.607	7.935
	Mesopores (10-100nm)	2.754	3.786	0.315	0.708	0.781	3.317	4.206	4.234
	Macropores (>100nm)	1.026	1.741	0.241	0.064	0.570	0.987	1.579	0.896
	Total	4.515	22.376	1.012	1.435	1.938	6.88	15.392	13.065

Supplementary Table 1 Results of N_2 gas isotherm adsorption of coal samples analyses

Sample ID	TCG	DG	PL	LY	SJZ	DS	XG	WTP
Langmuir volume (m^3/t)	42.78	47.26	27.19	27.47	39.03	52.61	35.6	48.14
Langmuir pressure (MPa)	1.47	3.68	0.71	0.68	0.68	1.21	2.37	1.09
Correlation coefficient	0.998	0.993	0.999	0.999	0.999	0.997	0.999	0.999

Supplementary Table 2 Results of CO2 isothermal adsorption analyses

Time after CO_2 injection (hour)		4	12	24	36	48	72
Reproducibility experiment-1	Absolute deviation (g)	0.0256	0.006	0.0381	0.0453	0.0095	0.0079
	Relative deviation (%)	20.32	2.72	14.35	15.33	2.71	2.10
Reproducibility experiment-2	Absolute deviation (g)	0.0096	0.0352	0.0217	0.0268	0.016	0.0263
	Relative deviation (%)	7.62	15.96	8.17	9.07	4.56	6.98

Supplementary Table 3 Absolute deviation and relative deviation between reproducibility experiment and series-B experiment at different CO2 residence time for sample SJZ

Time after CO_2 injection (hour)		4	12	24	36	48	72
Reproducibility experiment-1	Absolute deviation (g)	0.0701	0.021	0.0462	0.057	0.0256	0.0529
	Relative deviation (%)	27	4.31	7.041	7.92	3.33	6.52
Reproducibility experiment-2	Absolute deviation (g)	0.0800	0.0211	0.0367	0.0095	0.0092	0.007
	Relative deviation (%)	30.9	4.33	5.64	1.32	1.2	0.86

Supplementary Table 4 Absolute deviation and relative deviation between reproducibility experiment and series-B experiment at different CO2 residence time for sample DS

Supplementary Methods

N_2 adsorption at 77K

N_2 adsorption experiments at 77K were completed using an automated surface and volume analyzer MK ASAP-2020N following Chinese Oil and Industry Standard SY/T6154-1995. Coal samples captured between 60-80 mesh were dried in an oven at 80 ℃ overnight, and measured for the relative pressure range from 0.01 to 1 at 77K. N_2 gas isotherm adsorption experiments yield information on pores in the range 1.7 nm to 300 nm. The pore volume was calculated using BJH and the pore surface area was calculated using BET using the N_2 adsorption data.

CO_2 isothermal adsorption

CO_2 isothermal adsorption experiments were conducted for 8 samples following the Chinese GB/T 19560–2004 procedure using a TerraTek Isotherm Measurement System (ISO–300). The coal samples were crushed to powder at 60–80 mesh size and at weights up 80-100 g for moisture-equilibrium treatment. The samples were treated (ASTM standard) for at least four days until each sample weight stabilized. After these pretreatments, coal powder was placed into the sample cell of the ISO–300 for adsorption isotherm measurements. The adsorption experiments were carried out at constant temperature of 25 ℃, with pressure up to 4.9 MPa, at which CO_2 remains gaseous.

NMR experimental arrangement

Supplementary Fig. S14 shows the schematic diagram of the experimental arrangement, consisting of a gas-water exchange system and a thermostatic bath. The thermostatic bath is designed to maintain a constant temperature, and the attemperator coating on the magnetic coil surface is designed to maintain temperature when T_2 spectra are measured. The gas-water exchange apparatus comprises a

high pressure sample cell and a high-pressure gas-delivery system. The sample cell is specially designed to be nonmetallic and nonmagnetic and is located in the magnet coil, which is used to hold CO_2/helium gas. To pressurize the sample and to maintain a constant pressure, a high-pressure gas line, including a booster pump, two gas sources, a pressure resistant vessel, a pressure-regulating valve and several actuator-driven valves, is used. The pressure vessel and pressure-regulating valve are designed to maintain a constant pressure in the sample cell. High-precision pressure transducers and a temperature transmitter monitor the gas pressure and temperature in the gas-delivery system. The NMR measurement apparatus is a MiniMR-60 spectrometer, manufactured by Niumag Corporation Ltd, China. The instrument uses a frequency of 23.15MHz, a magnetic field strength of 0.54 T and a magnet coil diameter of 60 mm.

NMR measurements

The Carr-Purcell-Meiboom-Gill (CPMG) sequence, a standard sequence for measuring transverse relaxation times (T_2), is used in this study. The measurement parameters are appropriately chosen to minimize error in the experiments [30]. The wait time (*TW*) is selected to be more than five times that of the relaxation time of the slowest relaxing component, in order to ensure a quantitative result, *TW*=3s [24]. CPMG sequences of 10,000 echoes are used, ensuring that the complete decay curve is recorded. The chosen echo spacing (*TE*) and number of echo-trains for the NMR experiment are 0.32 ms and 64, respectively. For a given *TW*, the T_2 spectra distribution moves to the right with increasing *TE*, thus, the *TE* is set to be as small as possible to avoid the loss of information on some small pores [20].
